# Tirzepatide, GIP(1-42) and GIP(1-30) display unique signaling profiles at two common GIP receptor variants, E354 and Q354

**DOI:** 10.3389/fphar.2024.1463313

**Published:** 2024-10-11

**Authors:** Tayla A. Rees, Benjamin J. Buttle, Zoe Tasma, Sung-Hyun Yang, Paul W. R. Harris, Christopher S. Walker

**Affiliations:** ^1^ School of Biological Sciences, The University of Auckland, Auckland, New Zealand; ^2^ Maurice Wilkins Centre for Molecular Biodiscovery, The University of Auckland, Auckland, New Zealand; ^3^ Headache Group, Wolfson Sensory Pain and Regeneration Centre, Institute of Psychiatry, Psychology and Neuroscience, King’s College London, London, United Kingdom; ^4^ School of Chemical Sciences, The University of Auckland, Auckland, New Zealand

**Keywords:** gastric inhibitory polypeptide, glucose-dependent insulinotropic polypeptide, tirzepatide, G protein-coupled receptor, GIP receptor variants, obesity, diabetes

## Abstract

Type 2 diabetes (T2D) and obesity are prevalent metabolic disorders affecting millions of individuals worldwide. A new effective therapeutic drug called tirzepatide for the treatment of obesity and T2D is a dual agonist of the GIP receptor and GLP-1 receptor. Tirzepatide is clinically more effective than GLP-1 receptor agonists but the reasons why are not well understood. Tirzepatide reportedly stimulates the GIP receptor more potently than the GLP-1 receptor. However, tirzepatide signaling has not been thoroughly investigated at the E354 (wildtype) or Q354 (E354Q) GIP receptor variants. The E354Q variant is associated increased risk of T2D and lower body mass index. To better understand GIP receptor signaling we characterized the activity of endogenous agonists and tirzepatide at both GIP receptor variants. Using Cos7 cells we examined wildtype and E354Q GIP receptor signaling, analyzing cAMP and IP_1_ accumulation as well as AKT, ERK1/2 and CREB phosphorylation. GIP(1-42) and GIP(1-30)NH_2_ displayed equipotent effects on these pathways excluding CREB phosphorylation where GIP(1-30)NH_2_ was more potent than GIP(1-42) at the E354Q GIP receptor. Tirzepatide favored cAMP signaling at both variants. These findings indicate that tirzepatide is a biased agonist towards Gα_s_ signaling and suggests it equally activates the wildtype and E354Q GIP receptor variants. We also observed differences between the pharmacology of the GIP receptor variants with endogenous peptides, which may help to explain differences in phenotype. These findings contribute to a comprehensive understanding of GIP receptor signaling, and will aid development of therapies combating T2D and obesity.

## 1 Introduction

Type 2 diabetes (T2D) is a common metabolic disorder characterized by high blood glucose levels, due to low insulin or insulin resistance and affects more than 500 million people worldwide (Gallwitz, 2022). T2D incidence increases with age and is strongly associated with the prevalence of obesity (Bhupathiraju and Hu, 2016). For those living with diabetes, the disease can reduce lifespan and significantly affect quality of life (DeFronzo et al., 2015). The prevalence and severity of diabetes, especially T2D, has stimulated significant interest in developing effective therapies to manage this disease.

One major target of T2D therapies have been the incretin hormones and their receptors, which can stimulate a decrease in blood glucose levels via an increase in insulin secretion. The incretin hormones include glucose-dependent insulinotropic polypeptide (GIP) and glucagon-like peptide-1 (GLP-1). Together these peptide hormones are responsible for 60%-70% of the insulin response after glucose intake in humans (Ahrén, 2012; Nauck et al., 1986; Hansen et al., 2016). However in T2D, the glucose-reducing effect of GIP, but not GLP-1, is diminished (Pacini and Ahrén, 2017). This apparent resistance to GIP has also been observed for other GIP receptor agonists and may explain why there has been historically less success therapeutically targeting the GIP receptor alone. However, there are currently a suite of new GIP receptor targeted therapeutics in development (Ahlqvist et al., 2013; Saxena et al., 2010; Lynn et al., 2003; Almind et al., 1998). This led to the hypothesis that GLP-1, but not GIP receptor agonists, would be effective diabetes treatments. Several approved anti-diabetic drugs, such as semaglutide and dulaglutide, are GLP-1 receptor (GLP-1R) agonists (Holst and Rosenkilde, 2020). Clinical candidates targeting the GIP receptor alone have also been explored, but typically display much lower efficacy than GLP-1R agonists (Knerr et al., 2020). However, the recently approved drug tirzepatide does not appear to follow this trend. Tirzepatide is a co-agonist of the GIP and GLP-1 receptor, which was designed to improve glycemic control and aid in weight loss for T2D and obesity (Rosenstock et al., 2021; Wadden et al., 2023). Interestingly, tirzepatide displays similar activity to native GIP at the GIP receptor, but has lower affinity and potency than GLP-1 at the GLP-1R, suggesting it favors GIPR signaling (Gasbjerg et al., 2023; Willard et al., 2020; Coskun et al., 2018). However, tirzepatide-stimulated signaling at the GIP receptor and common receptor variants has not been thoroughly investigated.

The GIP receptor is a G protein-coupled receptor (GPCR) that binds the endogenous agonist GIP(1-42) and a c-terminally truncated isoform GIP(1-30)NH_2_ (Gabe et al., 2020). GIP(1-42) is the most abundant isoform and is secreted from K cells in the gut after nutrient ingestion, whereas GIP(1-30)NH_2_ is reported to be expressed by intestinal K cells and pancreatic α-cells (Yanagimachi et al., 2016; Yanagimachi et al., 2017; Hansen et al., 2016; Fujita et al., 2010a; Fujita et al., 2010b; Lund et al., 2015). Although GIP(1-42) and GIP(1-30)NH_2_ reportedly display equal potency and affinity at the GIP receptor, there are reported differences in biology indicating a need to comprehensively compare their pharmacology to elucidate any differences (Seino et al., 2010). Naturally occurring variants of the GIP receptor are also described, including one with a glutamine at position 354 (E354Q), which has been implicated in insulin resistance and T2D (Mohammad et al., 2014). There is evidence that GIP(1-42) and GIP(1-30)NH_2_ have a longer residence time at the E354Q receptor compared to the wildtype GIP receptor (Gabe et al., 2020). The effect of E354Q on receptor on cAMP signaling is well characterized however, the impact on other signaling cascades is not clear. Additionally, although receptor recycling is reported to be inhibited, which affects cell surface expression and further signaling (Almind et al., 1998; Fortin et al., 2010; Mohammad et al., 2014; Gabe et al., 2020). Currently, tirzepatide activity has not been described at the E354Q GIP receptor variant. Given the increased risk of diabetes associated with this variant, it is crucial to investigate whether tirzepatide acts effectively at this variant of the GIP receptor to assess whether it is likely to be effective in individuals who have this variant.

The GIP receptor was initially reported to display equivalent binding of GIP(1-42) and GIP(1-30)NH_2_ in transfected Chinese hamster lymphoblast cells, and was characterized as Gα_s_-coupled, based on cAMP accumulation in the absence of Ca^2+^ influx (Volz et al., 1995). Subsequent studies have primarily investigated GIP receptor signaling using GIP(1-42) in endogenous receptor expressing cell lines or primary models. Signaling in these models may be confounded by the presence of multiple different GIP receptor isoforms or related receptors including GLP-1R, the GLP-2 receptor and the glucagon receptor (Almind et al., 1998; Nakayama et al., 2014; Harada et al., 2008; Drucker, 2013). Differences between GIP and GLP-1 receptor signaling are often explained by the ability of GLP-1R to couple both Gα_s_ and Gα_q_ proteins, whereas GIP receptor is believed to only signal through Gα_s_ (Hauge et al., 2017; Oduori et al., 2020b; Mayendraraj et al., 2022). However, GIP receptor has been shown to recruit Gα_q_, but this interaction was not consistently observed and downstream signaling has not been examined (Novikoff et al., 2021; Jones et al., 2021). Therefore, additional characterization is required to determine whether GIP receptor couples to G proteins beyond Gα_s_. Understanding how the GIP receptor signals remains important to elucidating the activity of endogenous agonists and therapeutic agonists such as tirzepatide. Therefore, in this study we characterized the signaling profiles of endogenous agonists GIP(1-42) and GIP(1-30)NH_2_ and the drug tirzepatide at two GIP receptor isoforms, wildtype and E354Q, for five signaling pathways potentially relevant to the physiological actions of GIP in metabolism.

## 2 Materials and methods

### 2.1 Materials

GIP(1-42) was purchased from Genscript (Piscataway, NJ), tirzepatide was purchased from Focus Bioscience Pty (St Lucia, QLD, Australia), GIP(1-30)NH_2_ was synthesized in-house as described in the Supplementary Material (Supplementary scheme S1; Supplementary Figures SC1-S2). Peptide sequences are outlined in Supplementary Figure S1. All compounds were diluted in sterile water as 1 mM solutions, aliquoted into Protein LoBind tubes and stored at −30°C, with freeze-thaw cycles limited.

### 2.2 Plasmids and constructs

The “wildtype” (WT) human GIP receptor E354 variant construct in pcDNA3.1+ was purchased from Gene Universal Ltd. (Newark, DE). To generate the E354Q variant, a set of complementary primers were designed using NEBaseChanger (https://nebasechanger.neb.com) with the primer sequences as follows: Forward sequence - CTG​GGT​GTC​CAC​CAG​GTG​GTG​TTT​G, reverse sequence–CAAACACCACCTGGTGGACACCCAG. Generation of human GIP receptor E354Q variant was performed using KAPA HiFi HotStart kit (KAPA Biosystems, MA, United States) as per manufacturer’s instructions. Briefly, 20 ng of the “wildtype” human GIP receptor (E354 variant) was added to 2x KAPA HiFi HotStart ReadyMix, 0.3 μM of forward and reverse primers and DNAse-free water to make a final reaction volume of 25 μL. Using a PCR thermocycler (Applied Biosystems PCR system 9700, ThermoFisher, CA, United States), the DNA was initially denatured at 95°C for 2 min, followed by 25 cycles of 98°C for 30 s, 66°C for 15 s and 72°C for 3.5 min. Following the PCR reaction, 1 μL Dpn1 was added, and the DNA transformed into XL.10-gold Ultracompetent *E. coli* (Agilent Technologies, CA, United States). Plasmid DNA was purified using the NucleoBond Xtra Maxi kit (Macherey-Nagel, Germany) and sequenced prior to use (Centre for Genomics, Proteomics and Metabolomics, University of Auckland).

### 2.3 Cos7 cell culture and transfection

Cos7 cells were cultured and transfected as previously described (Tasma et al., 2022; Bower et al., 2018). Briefly, Cos7 cells were cultured in DMEM supplemented with 8% fetal bovine serum (FBS) in a 37°C humidified incubator with 5% CO_2_ and seeded into 96-well SpectraPlates at a density of 20,000 cells per well 24 h prior to transfection. Cells were transiently transfected using polyethyleneimine (PEI) with 0.25 μg of plasmid DNA per well (WT human GIP receptor (E354 variant), human GIP receptor E354Q variant, or empty vector (pcDNA3.1+)) as previously described (Bailey and Hay, 2006). All plasmid sequences were verified prior to use.

### 2.4 cAMP measurement

cAMP accumulation in Cos7 cells transfected with the WT or E354Q GIP receptors was measured using the LANCE cAMP detection kit (PerkinElmer Life and Analytical Sciences, MA, United States) as described previously (Woolley et al., 2017). Transfected cells underwent a serum-starve at 37°C for 30 min in 50 μL of cAMP assay media (DMEM +0.1% Bovine Serum Albumin (BSA) and 1 mM 3-isobutyl-1-methylxanthine (IBMX)) before stimulation. GIP (1-42), GIP (1-30), and tirzepatide were serially diluted in cAMP assay media. cAMP assay media alone or containing peptides were added to the cells and incubated at 37°C for 0-30 min. Following stimulation, all media was aspirated and replaced with 30 μL of ice-cold absolute ethanol, and the plates were stored at −20°C for a minimum of 10 min. The ethanol was evaporated and replaced with 50 μL of cAMP lysis buffer. Samples were shaken for 15 min before 5 μL of cell lysate was transferred to a white 384-well OptiPlate and processed for cAMP quantification as described previously (Walker et al., 2018). Samples were read using an Envision plate reader (PerkinElmer) and cAMP concentrations were determined from a standard curve generated in each assay.

### 2.5 IP_1_ measurement

The IP-one Gq assay kit (Cisbio, PerkinElmer) was used to quantify accumulated myo-inositol-1-phosphate (IP_1_), a by-product of IP3 produced after receptor-mediated Gα_q_ activation in Cos7 cells, as previously described (Tasma et al., 2022; Bower et al., 2018). Transfected cells underwent a serum-starve at 37°C for 30 min in 50 μL of serum-free media (DMEM +0.1% BSA) before stimulation. GIP (1-42), GIP (1-30), and Tirzepatide were serially diluted into assay media (DMEM +0.1% BSA +1% 10 mM LiCl). Fifty microliters of diluted peptide or media alone were added to the cells and incubated for 90 min at 37°C. Following this, the well contents were removed and replaced with 14 μL of IP-one Gq assay kit stimulation buffer to extract IP_1_. Samples were processed as previously described and read using an Envision plate reader (PerkinElmer). IP_1_ concentrations were determined from a standard curve generated in duplicate.

### 2.6 Measurement of phosphorylated AKT, ERK and CREB

Phosphorylated (p) Protein Kinase B (AKT), extracellular signal-regulated kinase 1/2 (ERK1/2) and CREB were detected using the AlphaLISA SureFire Ultra pAKT (Ser473), AlphaLISA SureFire Ultra pERK1/2 (Thr202/Tyr204) or AlphaLISA SureFire Ultra pCREB (Ser133) assay kit (PerkinElmer) as per the manufacturer’s protocol. Briefly, Cos7 cells were serum-starved in assay media (DMEM +0.1% BSA) for 4 h at 37°C with 5% CO_2_ prior to peptide stimulation. Peptides were serially diluted in assay media, and cells were incubated with assay media alone or each concentration of peptide for 15 min. FBS (50%) in pERK1/2 and pAKT or 50 μM forskolin in pCREB assays were used as positive controls. Media was then aspirated, and the cells were lysed in 40 μL of the kit lysis buffer, followed by shaking for 10–15 min at room temperature. Ten microliters of cell lysate was transferred to a white 384-well OptiPlate. Five microliters of acceptor beads coated with a Captsure tag immobilizing an ERK1/2, AKT, or CREB-specific antibody was added and incubated at room temperature in the dark for 1 h. Five microliters of donor beads coated with streptavidin, which captures a biotinylated antibody specific for the phosphorylated protein, was added and incubated in the dark at room temperature for 1 h. Plates were read on an Envision plate reader (PerkinElmer). In these assays, the signal is directly proportional, and so no standard curve was used.

### 2.7 Experimental design and data analysis

All data were plotted and analyzed using GraphPad Prism 8.0 (GraphPad Software Inc.). Data shown are the means ± standard error of the mean (s.e.m.) from n independent experiments, combined. In all experiments, the agonist positions were randomized in blocks on 96-well plates between independent experimental replicates. Each independent experimental replicate was performed with two or three technical repeats. Experimental replicates involved plating cells from a distinct passage, separate transient transfection and separate signaling assays which constituted an experimental n. Group sizes varied between n = 3 and n = 5. Concentration–response data were expressed as a percentage of the curve fitted maximum (E_max_) and minimum (E_min_) responses produced by GIP(1-42). E_max_ values were derived from raw, non-normalized values (cAMP and IP_1_) or were normalized and expressed as a percentage of the control GIP(1-42) E_max_ (pAKT, pERK1/2 and pCREB).

#### 2.7.1 Agonist assays

To define agonist potency, concentration-response curves were fitted with a four-parameter logistic equation. F tests were performed to determine whether the Hill slope of the curve differed significantly from one. In the majority of cases, the Hill slope was not significantly different from 1, the curves were constrained to 1, and pEC_50_ values obtained. If the Hill slope was not equal to one for the majority of independent experiments in a dataset, this parameter was unconstrained, and pEC_50_ values obtained.

#### 2.7.2 Quantifying relative efficacy and biased agonism

The data were fitted using an operational model of agonism added to GraphPad Prism to account for changes in agonist E_max_ and pEC_50_ (van der Westhuizen et al., 2014). For each signaling molecule, the E_max_ was constrained to the maximal normalized response by any agonist across the entire dataset and n was set to 1. Data were fitted as partial agonists to derive log (τ/K_A_) values (transduction coefficients). Log (τ/K_A_) represents a single value of efficacy that accounts for differences in both the maximal response and potency. Thus, relative efficacy comparisons allow more robust assessment of differences than comparisons of the individual E_max_ and pEC_50_ values. The SEM was calculated for the log (τ/K_A_) values generated from each individual experiment. Relative efficacy (Δlog (τ/K_A_)) and their error was then calculated by subtracting the reference agonist log (τ/K_A_) (GIP(1-42)) from each agonist log (τ/K_A_) value. Biased agonism values (ΔΔlog (τ/K_A_)) and their error were calculated using the agonist relative efficacy and by making between pathway comparisons for an agonist relative to a reference signaling molecule, cAMP (van der Westhuizen et al., 2014). ΔΔlog (τ/K_A_) values were determined by subtracting the relative efficacy (Δlog (τ/K_A_)) of an agonist at one signaling molecule from the relative efficacy of the same agonist for cAMP accumulation. The inverse log of the agonist Δlog (τ/K_A_) and ΔΔlog (τ/K_A_) values were used to create relative efficacy and bias radial plots which are presented.

### 2.8 Data and statistical analysis

For signaling data, pEC_50_ values from independent experimental replicates were derived from non-normalized data and combined. Significant differences in E_max_ were determined using a paired approach on the non-normalized agonist E_max_ values to generate error on the control agonist E_max_ and allow statistical significance to be determined (Kenakin, 2014). A ratio paired t-test on the raw data was used to compare E_max_ values between receptors and a ratio paired one-way ANOVA with *post hoc* Dunnett’s test using the log-transformed raw data was used to compare E_max_ comparisons between peptides at the same receptor. Statistical analysis of relative efficacy and biased agonism was performed on the Δlog (τ/K_A_) and ΔΔlog (τ/K_A_) values using a one-way ANOVA followed by a *post hoc* Dunnett’s test. In all cases, statistical significance was defined as *p* < 0.05.

## 3 Results

Initial experiments were conducted to confirm that receptor transfected Cos7 cells were an appropriate model to study GIP signaling. Neither GIP(1-42), GIP(1-30)NH_2_, nor tirzepatide stimulated cAMP accumulation in Cos7 cells transfected with vector alone (pcDNA3.1+) (Supplementary Figure S2). This suggests that Cos7 cells lack an endogenous functional GIP-responsive receptor and are therefore a suitable model to study GIP receptor signaling. Time course experiments were then undertaken to determine the optimal time to conduct the concentration-response experiments in GIP receptor transfected cells. Fifteen minutes of agonist stimulation was selected to determine cAMP accumulation and phosphorylation of AKT, ERK1/2 and CREB (Supplementary Figure S3). IP_1_ accumulation assays were performed at 90 min based on previous experiments (Tasma et al., 2022; Bower et al., 2018).

### 3.1 Agonist-stimulated signaling at the WT GIP receptor

In the global population, glutamine (E) is the most common residue reported at position 354 of the GIP receptor and was therefore considered the reference sequence, or wildtype (WT) form of the GIP receptor for this research (Kizilkaya et al., 2024). The ability of the recently approved tirzepatide with the two endogenous forms of GIP, GIP(1-42) and GIP(1-30)NH_2_, to activate the WT human GIP receptor was compared for five signaling pathways, cAMP, IP_1_, AKT, ERK1/2, and CREB. GIP(1-42) was used as the reference agonist in this research.

**FIGURE 1:**
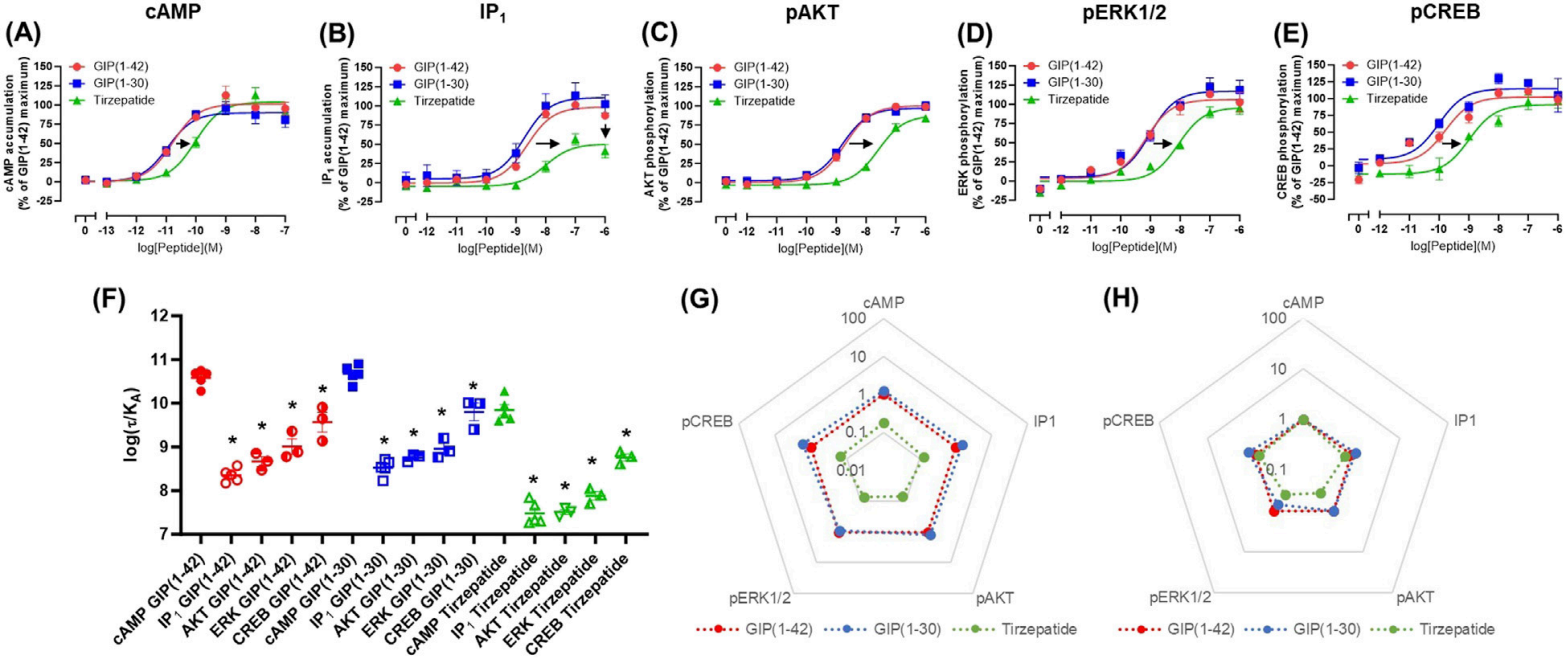
Intracellular signaling by GIP(1-42), GIP(1-30)NH_2_ and Tirzepatide at the human WT GIP receptor in transfected Cos7 cells **(A-E)**. Data were normalized to the maximal response produced by GIP(1-42) for each signaling pathway and expressed as a percentage. Arrows indicate a significant shift in potency or E_max_ relative to GIP(1-42). **(F)** Comparison of each peptide’s ability to induce signaling for different pathways at the WT GIP receptor. (G) Relative efficacy plot of Δlog (τ/KA) values, where GIP(1-42) is the reference peptide. **(H)** Web of bias plot of ΔΔlog (τ/KA) values, where the reference peptide is GIP(1-42) and the reference pathway cAMP. Data points are the mean ± s. e.m of the combined data from 3 (pAKT, pERK1/2 and pCREB) or 5 (cAMP and IP1) independent experiments. *p < 0.05 by one-way ANOVA with Dunnett’s *post hoc* test comparing the log (τ/KA) for each signaling pathway to cAMP for each peptide.

cAMP and IP_1_ accumulation along with phosphorylation of AKT, ERK and CREB are all key molecules involved in signaling events which promote insulin release and influence β-cell/adipocyte cell function and survival (Tengholm and Gylfe, 2017; Straub and Sharp, 1996; MacDonald et al., 2002; Song et al., 2007; Camaya et al., 2022; Ozaki et al., 2016; Khan et al., 2020). At the WT GIP receptor, both endogenous agonists GIP(1-42) and GIP(1-30)NH_2_, and tirzepatide stimulated a concentration-dependent increase in signaling for all pathways measures (Figures 1A–E). For cAMP, GIP(1-42) and GIP(1-30)NH_2_ produced an equipotent response while tirzepatide was ∼8-fold less potent (Figure 1A; Table 1). A similar profile was observed for IP_1_ accumulation with ∼4-fold lower potency for tirzepatide than GIP(1-42) (Figure 1B; Table 1). However, the E_max_ for tirzepatide was significantly lower than GIP(1-42), only reaching 54.9% ± 6.4% of the maximal response, indicative of a partial agonist (Table 1). Measurement of AKT, ERK1/2 and CREB phosphorylation exhibited a similar profile to cAMP accumulation, whereby GIP(1-42) and GIP(1-30)NH_2_ were equipotent, and significantly more potent than tirzepatide (Figures 1C–E; Table1). No differences in agonist E_max_ were observed for AKT, ERK1/2 or CREB phosphorylation. Non-normalized E_min_ and E_max_ values are summarized in Supplementary Table S1.

**TABLE 1:** Summary of GIP(1-42), GIP(1-30)NH_2_ and Tirzepatide potency and Emax values at the human WT and E354Q GIP receptors in transfected Cos7 cells.

GIP receptor	Peptide	cAMP		IP_1_		pAKT		pERK1/2		pCREB	
		pEC_50_	E_max_ (nM)	pEC_50_	E_max_ (nM)	pEC_50_	E_max_ (%)	pEC_50_	E_max_ (%)	pEC_50_	E_max_ (%)
WT	GIP(1-42)	10.8 ± 0.12	34.2 ± 7.75	8.62 ± 0.09	245 ± 31.5	8.72 ± 0.11	100 ± 0.13	9.53 ± 0.15	107 ± 3.23	10.0 ± 0.25	104 ± 0.07
	GIP(1-30)NH_2_	10.9 ± 0.07	32.3 ± 8.96	8.79 ± 0.10	263 ± 50.0	8.83 ± 0.12	97.1 ± 4.56	9.56 ± 0.20	117 ± 3.91	10.2 ± 0.12	117 ± 8.75
	Tirzepatide	9.88 ± 0.04^&^	33.5 ± 8.45	7.99 ± 0.13^&^	191 ± 18.1^&^	7.56 ± 0.03^&^	87.7 ± 2.60	8.61 ± 0.14^&^	96.3 ± 3.81^&^	9.01 ± 0.04^&^	91.5 ± 10.9
E354Q	GIP(1-42)	10.9 ± 0.18	35.5 ± 8.91	9.53 ± 0.17*	178 ± 26.4^#^	9.02 ± 0.03	56.6 ± 4.04^#^	9.50 ± 0.04	79.1 ± 8.69	10.6 ± 0.30	70.8 ± 26.9
	GIP(1-30)NH_2_	11.1 ± 0.06	32.8 ± 7.78	9.28 ± 0.10*	186 ± 24.3^#^	9.09 ± 0.03	50.9 ± 1.72^#^	9.66 ± 0.16	73.9 ± 9.75	11.5 ± 0.37*	70.7 ± 32.9
	Tirzepatide	9.96 ± 0.11^&^	32.5 ± 7.88	8.41 ± 0.17^&^	167 ± 21.4^#^	7.73 ± 0.12^&^	51.9 ± 8.95^#^	8.41 ± 0.30^&^	80.2 ± 17.3	9.29 ± 0.20^&^	59.4 ± 34.8

Data are the mean ± s. e.m of the combined data from 3 (pAKT, pERK1/2, pCREB) or 5 (cAMP, IP_1_) independent experiments. E_max_ values for cAMP, and IP_1_ are expressed in nM, E_max_ values for pAKT, pERK1/2 and pCREB, were normalized to GIP(1-42) at the WT GIP, receptor and expressed as a percentage. *p < 0.05 by unpaired Student’s t-test comparing the pEC_50_ of the same peptide at the WT, and E354Q GIP, receptor for each signaling pathway. ^&^ *p* < 0.05 by one-way ANOVA, with Dunnett’s *post hoc* test comparing the pEC_50_ or ratio paired one-way ANOVA, with Dunnett’s *post hoc* test comparing the log-transformed raw E_max_ values of each peptide to GIP(1-42) at the WT, or E354Q receptor for all signaling pathways. #*p* < 0.05 by ratio paired t-test comparing the raw E_max_ values of the same peptide at the WT, and E354Q GIP, receptor for each signaling pathway.

As tirzepatide exhibited apparent partial agonist activity for IP_1_ accumulation, all data were fitted using the Operational model of agonism (van der Westhuizen et al., 2014) to generate agonist transduction coefficients (log (τ/K_A_)) to examine any difference in potency when taking into consideration agonist E_max_. Compared to cAMP accumulation, all three agonists had transduction coefficients that were significantly lower for IP_1_, pAKT, pERK and pCREB, suggesting that these agonists favored cAMP signaling over all other pathways tested (Figure 1F; Table 2). The agonist relative efficacy (Δlog (τ/K_A_)) was then examined to determine if there were any agonist-dependent differences in pathway activations compared to the reference agonist GIP(1-42). Compared to GIP(1-42), GIP(1-30)NH_2_ displayed similar ability to stimulate cAMP and IP_1_ accumulation, and AKT, ERK and CREB phosphorylation, whereas tirzepatide displayed consistently lower relative efficacy (5 to 15-fold) (Figure 1G; Supplementary Table S2). To quantify whether the GIP receptor agonists displayed a preference for a specific signaling pathway at the WT GIP receptor, biased agonism (ΔΔlog (τ/K_A_)) was calculated using GIP(1-42) and cAMP as the reference agonist and pathway. Compared to cAMP accumulation, no agonists exhibited any significant bias towards or away from IP_1_, pAKT, pERK1/2, or pCREB (Figure 1H; Supplementary Table S2).

**TABLE 2:** Summary of peptide efficacy (log ((τ/KA)) values at WT and E354Q GIP receptors in transfected Cos7 cells.

GIP receptor	Peptide	Log (τ/K_A_)				
		cAMP	IP_1_	pAKT	pERK1/2	pCREB
WT	GIP(1-42)	10.6 ± 0.09	8.35 ± 0.07^	8.67 ± 0.11^	9.01 ± 0.18^	9.57 ± 0.23^
	GIP(1-30)NH_2_	10.7 ± 0.09	8.53 ± 0.08^	8.76 ± 0.05^	8.96 ± 0.13^	9.80 ± 0.20^
	Tirzepatide	9.85 ± 0.12^&^	7.48 ± 0.12^&^^	7.51 ± 0.06^&^^	7.88 ± 0.10^&^^	8.76 ± 0.08^&^^
E354Q	GIP(1-42)	10.7 ± 0.18	9.24 ± 0.10*^	8.98 ± 0.02^	9.22 ± 0.14^	10.2 ± 0.24
	GIP(1-30)NH2	10.9 ± 0.03*	9.10 ± 0.03*^	8.99 ± 0.26^	9.45 ± 0.26 ^	11.3 ± 0.41*
	Tirzepatide	9.83 ± 0.06^&^	8.07 ± 0.14*^&^^	7.66 ± 0.14^&^^	7.92 ± 0.08^&^^	8.94 ± 0.13^&^^

Data are mean ± s. e.m of the combined data from 3 (pAKT, pERK1/2, pCREB) or 5 (cAMP, IP_1_) independent experiments. The operational model was performed on data normalized to the maximal response produced by GIP(1-42) for each receptor. *p < 0.05 by unpaired Student’s t-test comparing the log ((τ/K_A_) of the peptide at the E354Q GIP, receptor with the WT GIP, receptor for each signaling assay. ^&^ *p* < 0.05 by one-way ANOVA, with Dunnett’s *post hoc* test comparing the log (τ/K_A_) for each peptide to GIP(1-42) at that receptor. ^*p* < 0.05 by one-way ANOVA, with Dunnett’s *post hoc* test comparing the log (τ/KA) for each signaling pathway to cAMP, for each peptide at that receptor.

### 3.2 Agonist-stimulated signaling at the E354Q GIP receptor

**FIGURE 2:**
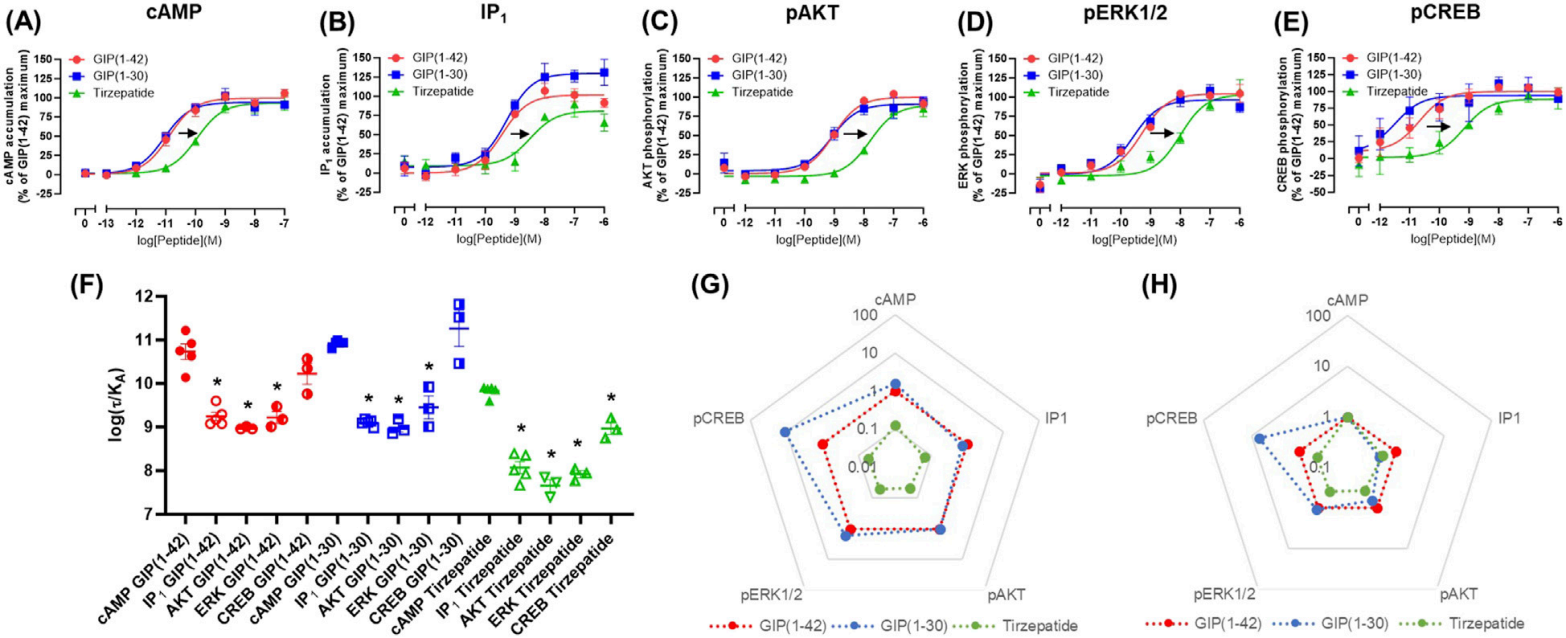
Intracellular signaling by GIP(1-42), GIP(1-30)NH_2_ and Tirzepatide at the human E354Q GIP receptor in transfected Cos7 cells **(A-E)**. Data were normalized to the maximal response produced by GIP(1-42) for each signaling pathway and expressed as a percentage. Arrows indicate a significant shift in potency or E_max_ relative to GIP(1-42). **(F)** Comparison of the ability of each of the peptides to induce signaling for different pathways at the E354Q GIP receptor. **(G)** Relative efficacy plot of Δlog (τ/KA) values for the E354Q GIP receptor, where GIP(1-42) is the reference peptide. **(H)** Web of bias plot of ΔΔlog (τ/KA) values for the E354Q GIP receptor, where the reference peptide is GIP(1-42) and the reference pathway cAMP. Data points are the mean ± s. e.m of the combined data from 3 (pAKT, pERK1/2 and pCREB) or 5 (cAMP and IP1) independent experiments. *p < 0.05 by one-way ANOVA with Dunnett’s *post hoc* test comparing the log (τ/KA) for each signaling pathway to cAMP for each peptide.

At the E354Q GIP receptor, GIP(1-42), GIP(1-30)NH_2_ and tirzepatide all stimulated concentration-dependent signaling for all pathways measured (Figures 2A–E). GIP(1-42) and GIP(1-30)NH_2_ equipotently stimulated cAMP accumulation, while tirzepatide was ∼9-fold less potent (Figure 1A; Table 1). IP_1_ accumulation displayed a similar agonist profile to cAMP accumulation, with tirzepatide being ∼13-fold less potent than GIP(1-42) (Figure 2B; Table 1). There were no significant differences in IP_1_ E_max_ observed for the three agonists (Table 1). For pAKT, pERK1/2 and pCREB agonist profiles GIP(1-42) and GIP(1-30)NH_2_ were approximately equipotent and significantly more potent than tirzepatide (Figures 2C–E; Table 1). No significant differences in E_max_ were observed for AKT, ERK1/2 or CREB phosphorylation. Non-normalized E_min_ and E_max_ values are summarized in Supplementary Table S1.

In order to consider the contribution of both agonist potency and E_max_ to differences in agonist activity, transduction coefficients (log (τ/K_A_) were determined. For GIP(1-42), GIP(1-30)NH_2_ and tirzepatide, the transduction coefficients for cAMP accumulation were significantly higher than for IP_1_, pAKT and pERK1/2 (Figure 2F; Table 2). Interestingly, for pCREB the transduction coefficient of GIP(1-42) and GIP(1-30)NH2, but not tirzepatide, were not significantly different to those for cAMP (Table 2). Relative to GIP(1-42), GIP(1-30)NH_2_ had a similar efficacy (Δlog (τ/K_A_)) induction of cAMP accumulation, pAKT and pERK (Figure 2G). For pCREB, GIP(1-30)NH_2_ was ∼11-fold more efficacious compared to GIP(1-42); however, this did not reach significance (Supplementary Table S2). Tirzepatide consistently exhibited lower efficacy relative to GIP(1-42) for all five signaling pathways (8 to 21-fold) (Figure 2G). Limited biased agonism (ΔΔlog (τ/K_A_)) was observed at the E354Q GIP receptor (Figure 2H) GIP(1-30)NH_2_ appeared to be biased away from IP_1_ (2-fold) and towards pCREB (7-fold) compared to cAMP accumulation, although these did not reach significance (Supplementary Table S2). Similarly, tirzepatide appeared to be biased away from pAKT, pERK and pCREB (2.6, 2.5 and 2.3-fold, respectively) compared to cAMP, but these differences were not significant (Supplementary Table S2).

### 3.3 Comparison of agonist stimulation of the wildtype and E354Q GIP receptor variants across multiple pathways

**FIGURE 3:**
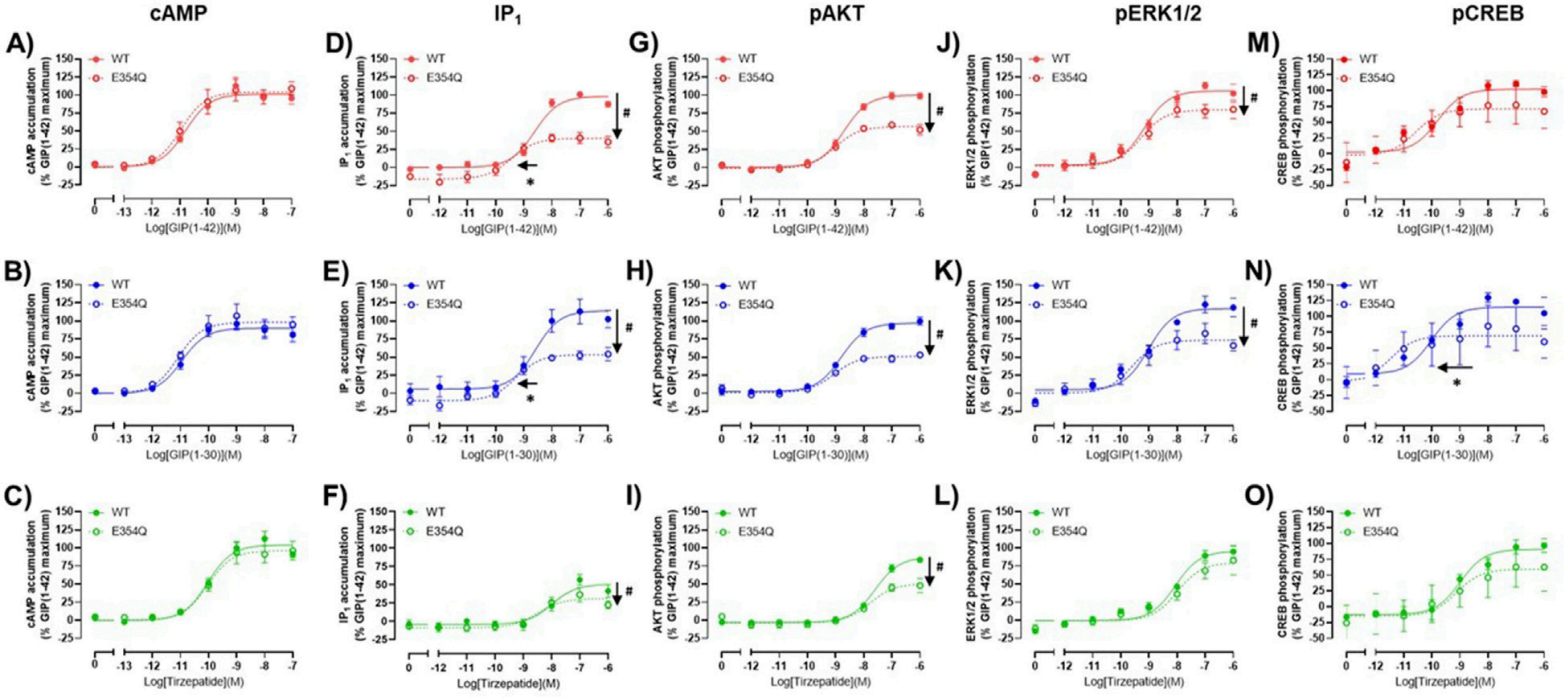
Comparison of GIP(1-42), GIP(1-30)NH_2_ and Tirzepatide activation of intracellular signaling at the human WT and E354Q GIP receptors in transfected Cos7 cells **(A–O)**. To allow direct comparisons between the receptors, all data for a signaling pathway were normalized to the maximal response produced by GIP(1-42) at the WT GIP receptor. Data points are the mean ± s. e.m of the combined data from 3 (pAKT, pERK1/2 and pCREB) or 5 (cAMP and IP1) independent experiments. *p < 0.05 by unpaired Student’s t-test comparing the pEC_50_ of the same peptide at the WT and E354Q GIP receptor for each signaling assay. #*p* < 0.05 by unpaired t-test comparing the E_max_ of the same peptide at the WT and E354Q GIP receptor for each signaling pathway.

The pharmacological profiles of the WT and E354Q GIP receptor variants were compared to investigate whether there were differences between agonist signaling profiles. To allow comparison, data were normalized to GIP(1-42) at the WT receptor for each signaling pathway. GIP(1-42), GIP(1-30)NH_2_ and tirzepatide exhibited similar cAMP activation at the WT and E354Q receptors, with no observed difference in potency or maximal response (Figures 3A–C). For IP_1_ accumulation, GIP(1-42) and GIP(1-30)NH_2_ were significantly more potent at the E354Q receptor compared to the WT receptor (Figures 3D, E; Table 1). Additionally, the E_max_ values of all three peptides were lower at the E354Q receptor compared to the WT receptor. AKT and ERK phosphorylation displayed no differences in agonist potency between the receptors (Figures 3G–L). However, the maximal pAKT and pERK were significantly lower at the E354Q variant compared to the WT receptor for GIP(1-42) and GIP(1-30)NH2. The E_max_ for tirzepatide-stimulated pAKT, but not pERK1/2, was lower at the E354Q receptor (Table 1). There were no observed differences in potency for CREB phosphorylation at the E354Q and WT GIP receptor variants for GIP(1-42) and tirzepatide (Figures 3M–O; Table 1). In contrast, GIP(1-30)NH_2_ was ∼20-fold more potent at the E354Q variant compared to the WT receptor (Figure 3N Table 1). A trend of the E354Q variant displaying a lower maximal induction of agonist-stimulated CREB phosphorylation than at the WT receptor was observed for all three agonists, however, this was not significant.

**FIGURE 4:**
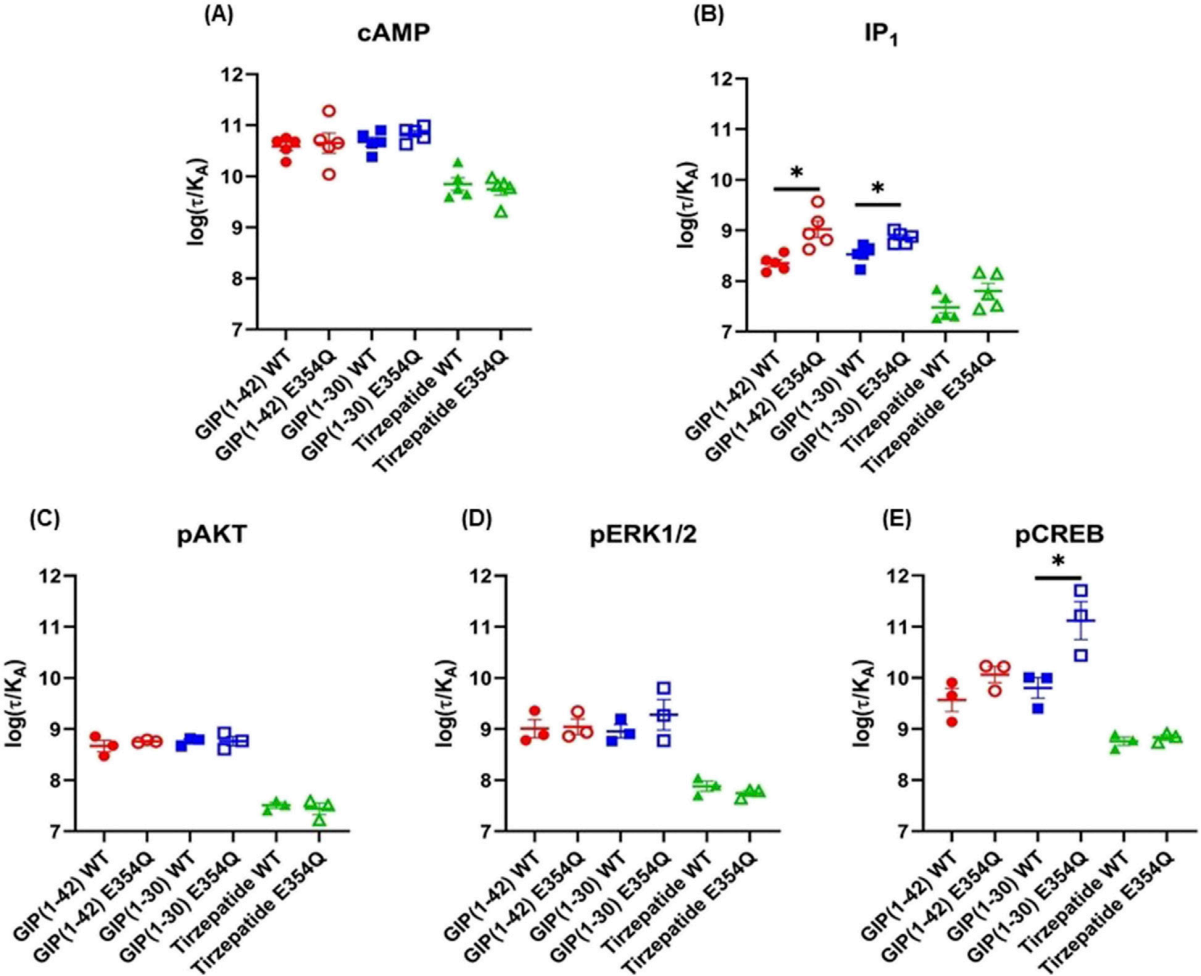
Comparison of GIP(1-42), GIP(1-30)NH_2_ and Tirzepatide efficacy (log(τ/KA)) at the human WT and E354Q GIP receptors in transfected Cos7 cells **(A-E)**. To allow direct comparisons between the receptors the operational model was performed on data normalized to the maximal response produced by GIP (1-42) at the WT GIP receptor, for both the WT and E354Q GIP receptor. Data points are the mean ± s.e.m of the combined data from 3 (pAKT, pERK1/2 and pCREB) or 5 (cAMP and IP1) independent experiments. *p < 0.05 by unpaired Student’s t-test comparing the log (τ/KA) of the same peptide at the WT and E354Q GIP receptors for each signaling pathway.

The Operational model of agonism was employed to consider the contribution of both potency and E_max_ to differences in signaling. Consistent with the observed pEC_50_ values, GIP(1-42) and GIP(1-30)NH_2_ had greater transduction coefficients at the E354Q than WT receptor for IP_1_ accumulation. Furthermore, the transduction coefficient for GIP(1-30)NH_2_ was significantly greater at the E354Q than WT receptor for pCREB (Figure 4; Table 3).

**TABLE 3:** Comparison of GIP(1-42), GIP(1-30)NH_2_ and Tirzepatide efficacy (log (τ/KA)) at human WT and E354Q GIP receptors, when normalized to GIP(1-42) at the WT GIP receptor.

GIP receptor	Peptide	Log (τ/K_A_)				
		cAMP	IP_1_	pAKT	pERK1/2	pCREB
WT	GIP(1-42)	10.6 ± 0.04	8.35 ± 0.07	8.67 ± 0.11	9.01 ± 0.18	9.57 ± 0.22
	GIP(1-30)NH_2_	10.7 ± 0.09	8.53 ± 0.08	8.76 ± 0.05	8.96 ± 0.13	9.80 ± 0.20
	Tirzepatide	9.85 ± 0.12	7.48 ± 0.11	7.51 ± 0.06	7.88 ± 0.10	8.76 ± 0.08
E354Q	GIP(1-42)	10.7 ± 0.20	9.02 ± 0.16*	8.76 ± 0.11	9.05 ± 0.15	10.1 ± 0.16
	GIP(1-30)NH_2_	10.8 ± 0.06	8.86 ± 0.05*	8.77 ± 0.09	9.28 ± 0.30	11.1 ± 0.37*
	Tirzepatide	9.75 ± 0.11	7.80 ± 0.15	7.44 ± 0.11	7.75 ± 0.05	8.83 ± 0.05

Data normalized to the maximal response produced by GIP(1-42) at the WT GIP, receptor, for both the WT, and E354Q GIP, receptor. The data was then fitted to the Operational model. Data are mean ± s. e.m of the combined data from 3 (pAKT, pERK1/2, pCREB) or 5 (cAMP, IP_1_) independent experiments. *p < 0.05 by unpaired Student’s t-test comparing the log ((τ/K_A_) of the same peptide at the WT, and E354Q GIP, receptors for each signaling pathway.

## 4 Discussion

### 4.1 GIP receptors couple to Gα_s_ and Gα_q_

Understanding the signalling of GIP receptors is essential to elucidating their physiological effects and exploiting this receptor as a drug target. The GIP receptor is often reported to couple to Gα_s_ alone (Oduori et al., 2020b). However, we observed that GIP(1-42), GIP(1-30)NH_2_, and tirzepatide induce both cAMP and IP_1_ accumulation. This indicates potential coupling to Gα_q_, as IP_1_ is a stable by-product of IP_3_ (Garbison et al., 2004). The induction of cAMP accumulation by the endogenous GIP agonists was similar to previous studies (Hansen et al., 2016; Yuliantie et al., 2021). Tirzepatide binds to albumin, resulting in an extended half-life (Coskun et al., 2018). The lower potency we observed for tirzepatide than GIP(1-42) was potentially caused by binding and sequestration of trizepatide by BSA in the assay media. Reduced tirzepatide cAMP potency in the presence of BSA has been elegantly demonstrated (Willard et al., 2020). Therefore, BSA likely caused a conserved reduction in trizeaptide potency (pEC_50_) compared to GIP across all signalling molecules tested. However, the relative activation of different signalling pathways by tirzepatide will remain unaffected by the presence of BSA. There is mixed evidence of Gα_q_-coupling to the GIP receptor in the literature (Jones et al., 2021; Novikoff et al., 2021; Manchanda et al., 2023). Interpretation of these studies is complicated by the use of modified receptors and signalling proteins, which can affect pharmacology and signalling behaviour (Bonneterre et al., 2016). Our data using unmodified receptors and endogenous signalling machinery supports the coupling of GIP receptors to Gα_q_, with a preference of ∼100-fold for Gα_s_ over Gα_q_. Furthermore, we observed the activation of signalling molecules that can be downstream of multiple G proteins, including Gα_s_, Gα_q_ and Gβγ (Mayendraraj et al., 2022; Elghazi et al., 2007; Khan et al., 2013; Ehses et al., 2002). GIP receptors have previously been shown to activate these phosphorylated signalling molecules in a variety of physiologically relevant cell lines, including INS-1 and 3T3-L1 cells (Kim et al., 2005; Widenmaier et al., 2010; Kim et al., 2008; Kim et al., 2010). Interestingly, observed activation of AKT, ERK1/2 and CREB phosphorylation was ∼10-50-fold more potent than previous reports (Yuliantie et al., 2020; Yuliantie et al., 2021; Kim et al., 2005; Kim et al., 2008; Ehses et al., 2002). This could be due to different cellular backgrounds or receptor overexpression, compared to endogenous expression in the physiologically relevant cell lines.

### 4.2 The E354Q variant displays differences in signalling compared to the “WT” GIP receptor

The single nucleotide GIPR receptor polymorphism (rs1800437) is associated with a lower BMI and less susceptibility to obesity. This is due to an amino acid substitution from glutamine to glutamic acid at position 354 (E354Q) of the GIP receptor (Vogel et al., 2009; Saxena et al., 2010; Berndt et al., 2013). Similarly, mice engineered to express this polymorphism display greater sensitivity to GIP, glucose tolerance, and a lean phenotype (Yammine et al., 2023). Differences in internalisation between the “wildtype” E354 and E354Q GIP receptor variants have been reported (Yammine et al., 2023; Gabe et al., 2020; Mohammad et al., 2014; Abdullah et al., 2016; Willard et al., 2020). However, signalling events underlying this stark difference in metabolism are currently unclear. Therefore, we comprehensively compared the induction of potentially relevant signalling molecules between the two variants. Herein, we report the first differences in signaling between the two variants. Consistent with previous studies, no major differences were observed stimulating cAMP accumulation at either variant (Gabe et al., 2020; Almind et al., 1998; Mohammad et al., 2014; Kubota et al., 1996). However, maximal levels of IP_1_ accumulation, AKT phosphorylation and ERK phosphorylation were significantly lower for the E354Q variant. Interestingly, no significant difference in E_max_ was observed for either cAMP accumulation or CREB phosphorylation, suggesting that lower E_max_ is not universal and receptor expression is unlikely to be a factor. However, we cannot entirely rule out that this reduction in E_max_ may be linked to differences in efficiency of receptor expression (Kunath et al., 2003). Therefore, future studies comparing these receptors should also consider quantifying and comparing the expression of the receptors. Interestingly, GIP(1-42) and GIP(1-30)NH_2_ displayed a small but significant increase in potency, and therefore, efficacy for IP_1_ accumulation at the E354Q variant. This suggests greater induction of Gα_q_ coupling and signaling at the E354Q variant. Given that Gα_q_ signaling cascades contribute to calcium influx, a key step in secretory vesicle release, more efficacious Gα_q_ signaling could increase insulin exocytosis (Thore et al., 2005; Thompson and Kanamarlapudi, 2015; Oduori et al., 2020a). This is the first study to compare the efficacy of tirzepatide at common variants of the human GIP receptor. Tirzepatide signaling was predominantly the same between the two receptor variants. However, there were subtle differences, including lower maximal stimulation of IP_1_ accumulation and AKT phosphorylation at the E354Q variant. These minor differences are unlikely to effect the efficacy of this drug. Overall this suggests that assuming there are no major differences in GIP receptor variant expression, tirzepatide likely has equivalent function in people expressing either the wildtype or E354Q GIP receptor variants.

### 4.3 Biased agonism

The GIP receptor is reported to be biased towards Gα_s_ coupling (Yuliantie et al., 2020; Willard et al., 2020). Interestingly, we observed the most pronounced biased agonism for tirzepatide. GIP and tirzepatide occupy the same general structural position when activating the GIP receptor. However, tirzepatide makes an additional hydrogen bond with and arginine residue (R190) in the GIP receptor, which is hypothesized to facilitate changes in G protein binding affinity and signaling (Sun et al., 2022). Interestingly, R190 by alanine is crucial for GIP-mediated cAMP accumulation (Yuliantie et al., 2021). Therefore, it is perhaps unsurprising that an interaction between R190 and tirzepatide favors Gα_s_ coupling and cAMP signaling. This is consistent with lower accumulation of IP_1_ and phosphorylation of AKT, ERK1/2, and to a lesser extent, CREB. Lower Gα_q_-associated activation these molecules could account for this profile of signaling. However, the links between some of these molecules and the Gα_q_ signaling are tenuous and further research is required to elucidate these connections (Baggio and Drucker, 2007; Mayendraraj et al., 2022). The lower E_max_ observed for tirzepatide was broadly consistent with a previous report. However, they reported bias towards ERK1/2 phosphorylation at both the GIP and GLP-1 receptors (Yuliantie et al., 2020). The reasons for this difference are unclear. However, differences in the compliment of intracellular proteins and the time-point selected for analysis may be important factors.

### 4.4 Physiological relevance to pancreatic β-cells and adipocytes

Given the emerging importance of GIP receptor targeted therapeutics in metabolic disease, understanding the role specific signalling pathways play in physiological processes is of significant interest. Circulating GIP may be sufficient to activate Gα_s_, but not Gα_q_, coupled signalling (Marathe et al., 2020). This suggests that G_q_ coupled signalling may be of greater significance at sites where GIP is released and local concentrations are higher. GIP released from pancreatic α cells could result in local concentrations sufficient to activate both Gα_s_ and Gα_q_ in pancreatic β-cells (El et al., 2021; Fujita et al., 2010b). Gα_s_ and Gα_q_ coupled signaling act in combination to enhance insulin release from pancreatic β-cells (Tengholm and Gylfe, 2017; Straub and Sharp, 1996; MacDonald et al., 2002). Downstream, the phosphorylation AKT and CREB been linked to changes in β-cell function and survival (Song et al., 2007; Camaya et al., 2022; Khan et al., 2020; Kim et al., 2008). We observed that GIP(1-30)NH_2_, displayed greater efficacy for CREB and IP_1_ activation at the E354Q GIP receptor variant. Bias towards these pathways would predictably lead to greater insulin release and thus improved metabolic health. However, increased activity at these pathways may contribute to the greater long-term downregulation which would not be reflected in the current experimental design. GIP receptor activation in adipocytes adds further complexity to understanding the metabolic phenotypes (Kagdi et al., 2024). Gα_s_-mediated cAMP accumulation appears to be the major pathway activated by GIP in adipocytes. However, GIP mediated activation of AKT and ERK1/2 are linked to adipocyte development and lipolysis, respectively (Song et al., 2007; Camaya et al., 2022; Ozaki et al., 2016). The precise profile of GIP receptor signaling likely depends upon other circulating factors present, and the expression of accessory and signaling proteins in an individual cell. Interestingly, Gα_q_ signaling is known to tightly regulate adipocyte differentiation and associated with greater triglyceride storage (Kongthitilerd et al., 2023; Shi et al., 2000). Therefore, therapeutically targeting Gα_q_ signaling could reduce adipocyte mass and explain observed weight loss phenotype with trizepatide.

### 4.5 Further considerations and future directions

Further experiments are required to elucidate the role Gα_q_ plays in GIP receptor and GLP-1 receptor signaling (Baggio and Drucker, 2007; Seino et al., 2010; Nauck et al., 2021). The notion that the difference in therapeutic utility between GLP-1 and GIP hinges on a lack of Gα_q_ coupling to the GIP receptor requires re-evaluation, given that the GIP receptor appears to couple Gα_q_. Other differences, such as the differential expression of GLP-1 and GIP receptors in adipocytes, the effect of accessory proteins and other signaling should be considered. Interestingly, both receptors have been shown to interact with receptor activity-modifying proteins (RAMPs) (Serafin et al., 2020; Lorenzen et al., 2019; Bower et al., 2018). Mouse models deficient in RAMP1 and RAMP3 display enhanced body weight reduction in response to both GIP and GLP-1 (Leuthardt et al., 2023). Whether these receptors are differentially modulated by RAMPs *in vivo* remains unclear.

## 5 Summary

This study described the complex signaling of GIP(1-30)NH_2_, GIP(1-42) and tirzepatide at the GIP receptor. Interestingly, The GIP receptor appeared couple Gα_s_ and, albeit relatively weakly, Gα_q_ signaling pathways. Furthermore, we showed that tirzepatide is a biased agonist towards Gα_s_ signaling and displayed similar activation of the wildtype and E354Q GIP receptor variants. Differences between the pharmacology of the GIP receptor variants may help to explain differences in the metabolic phenotypes observed and the effect of different GIP receptor agonists on metabolism. These findings contribute to a comprehensive understanding of GIP receptor signaling, and will aid the development of therapies combating T2D and obesity.

## Data Availability

The raw data supporting the conclusions of this article will be made available by the authors, without undue reservation.
