# Supplementary material for: Tirzepatide, GIP(1-42) and GIP(1-30) display unique signaling profiles at two common GIP receptor variants, E354 and Q354
Source: Front Pharmacol. 2024 Oct 11;15:1463313. doi: 10.3389/fphar.2024.1463313 (PMC11502443; doi:10.3389/fphar.2024.1463313)
Supplement: Supplementary file 1 [file DataSheet1.PDF]

Tirzepatide, GIP(1-42) and GIP(1-30) display unique signaling profiles at two common GIP receptor variants, E354 and Q354

Tayla Anne Rees[1,2,3]*, Benjamin John Buttle[1], Zoe Tasma[1,2], Sung-Hyun Yang[1], Paul W.R. Harris[1,2,4], Christopher Stuart Walker[1,2]*

[1]School of Biological Sciences, The University of Auckland, Auckland 1010, New Zealand

[2]Maurice Wilkins Centre for Molecular Biodiscovery, The University of Auckland, Auckland 1010, New Zealand

[3]Headache Group, Wolfson Sensory Pain and Regeneration Centre, Institute of Psychiatry, Psychology and Neuroscience, King's College London, London, UK

[4]School of Chemical Sciences, The University of Auckland, Auckland 1010, New Zealand

Correspondence:
*Tayla Rees; tayla.rees@kcl.ac.uk and Christopher Walker; cs.walker@auckland.ac.nz

Supplemental Methods

General Procedure

All reagents were purchased as reagent grade and used without further purification. All amino acids utilised in the synthesis adopt the (*L*)-configuration. *N*,*N*-Diisopropylethylamine (DIPEA), piperidine, *N*,*N′*-diisopropylcarbodiimide (DIC), 1,2-ethanedithiol (EDT) and triisopropylsilane (TIPS) were purchased from Sigma-Aldrich (St. Louis, Missouri). *O*-(7-Azabenzotriazol-1-yl)-*N*,*N*,*N′*,*N′*-tetramethyluronium hexafluorophosphate (HATU), Fmoc-Ala-OH, Fmoc-Asn(Trt)-OH (Trt = triphenylmethane), Fmoc-Asp(*t*Bu)-OH (*t*Bu = *tert*-butyl), Fmoc-Gln(Trt)-OH, Fmoc-Glu(*t*Bu)-OH, Fmoc-Gly-OH, Fmoc-His(Trt)-OH, Fmoc-Ile-OH, Fmoc-Leu-OH, Fmoc-Lys(Boc)-OH (Boc = *tert*-butoxycarbonyl), Fmoc-Met-OH, Fmoc-Phe-OH, Fmoc-Ser(*t*Bu)-OH, Fmoc-Thr(*t*Bu)-OH, Fmoc-Trp(Boc)-OH, Fmoc-Tyr(*t*Bu)-OH and Fmoc-Val-OH were purchased from GL Biochem (Shanghai, China). 4-[(2,4-Dimethoxyphenyl)(Fmoc-amino)methyl]phenoxyacetic acid (Rink amide linker) was purchased from CS Bio (Shanghai, China). 6-Chloro-1-hydroxybenzotriazole (6-Cl-HOBt) was purchased from Aapptec (Louisville, Kentucky). Aminomethyl TentaGel® S resin was purchased from Rapp Polymere (Tübingen, Germany). Yields refer to chromatographically homogeneous materials. Semi-preparative/analytical RP-HPLC was performed on a Thermo Scientific (Waltham, MA) Dionex Ultimate 3000 HPLC equipped with a four channel UV detector at 210, 225, 254 and 280 nm using either a Phenomenex analytical column (Torrance, CA), Gemini® C18 (5 µm; 4.6 × 150 mm) at a flow rate of 1.0 mL/min, or a Phenomenex semi-preparative column (Torrance, CA), Gemini® C18 (5 µm; 10 × 250 mm) at a flow rate of 4 mL/min. A suitably adjusted gradient of 5% B to 95% B was used for HPLC, where solvent A was 0.1% TFA in H_2O and B was 0.1% TFA in acetonitrile. LC-MS spectra were acquired using Agilent Technologies (Santa Clara, CA) 1260 Infinity LC equipped with an Agilent Technologies 6120 Quadrupole mass spectrometer. An Agilent C3 analytical column (3.5 µm; 3.0 × 150 mm) was used at a flow rate of 0.3 mL/min using a linear gradient of 5% B to 95% B over 30 min, where solvent A was 0.1% formic acid in H_2O and B was 0.1% formic acid in acetonitrile.

General Methods

Method 1: General procedure for attachment of Fmoc Rink amide to the resin:
To aminomethyl TentaGel® S resin (370 mg, 0.1 mmol, loading: 0.27 mmol/g) pre-swollen in CH_2Cl_2 (5 mL, 30 min), was added 4-[(2,4-dimethoxyphenyl)(Fmoc-amino)methyl]phenoxyacetic acid (Rink amide linker) (270.0 mg, 5 equiv., 0.5 mmol) and 6-Cl-HOBt (77.0 mg, 4.5 equiv., 0.45 mmol) dissolved in DMF (3.0 mL) followed by addition of DIC (78 µL, 5 equiv., 0.5 mmol). The reaction mixture was gently agitated at room temperature for 24 h. The resin was filtered and washed with DMF (3 × 3.0 mL) after which a negative ninhydrin test confirmed successful coupling**.**

Method 2: General procedure for removal of *N*$^{\alpha}$-Fmoc-protecting group:
Peptidyl resin was treated with a solution of 20% piperidine in DMF (4 mL, *v*/*v*) and the mixture was agitated at room temperature for 2 ×10 min. The resin was filtered and washed with DMF (3 × 3.0 mL).

Method 3: General coupling procedure for Fmoc-Ala-OH, Fmoc-Asn(Trt)-OH, Fmoc-Asp(*t*Bu)-OH, Fmoc-Gln(Trt)-OH, Fmoc-Glu(*t*Bu)-OH, Fmoc-Gly-OH, Fmoc-His(Trt)-OH, Fmoc-Ile-OH, Fmoc-Leu-OH, Fmoc-Lys(Boc)-OH, Fmoc-Met-OH, Fmoc-Phe-OH, Fmoc-Ser(*t*Bu)-OH, Fmoc-Thr(*t*Bu)-OH, Fmoc-Trp(Boc)-OH Fmoc-Tyr(*t*Bu)-OH and Fmoc-Val-OH:
Manual Couplings were performed with the appropriate Fmoc-protected amino acid (0.5 mmol, 5.0 equiv.), HATU (181 mg, 0.475 mmol, 4.75 equiv.) and DIPEA (210 µL, 1.2 mmol, 12 equiv.) in DMF (3.0 mL) for 45 min at room temperature. The resin was filtered and washed with DMF (3 × 3.0 mL).

Method 4: General procedure for TFA-mediated resin cleavage and global deprotection:

Peptidyl resin was treated with a mixture of TFA/H_2O/TIPS/EDT (91.5:5:2.5:1, *v/v/v/v*, 10 mL) for 120 min at room temperature. The resin was filtered, and the filtrate was partially concentrated under a gentle stream of N_2 gas, followed by the addition of cold diethyl ether to form a precipitate. The mixture was centrifuged, and the solution was carefully decanted and discarded. The diethyl ether wash was repeated twice before dissolving the solid pellet in H_2O:acetonitrile containing 0.1% TFA (1:1, *v/v,* 25 mL) and lyophilised.

Synthesis of GIP (1-30) using Fmoc-SPPS.

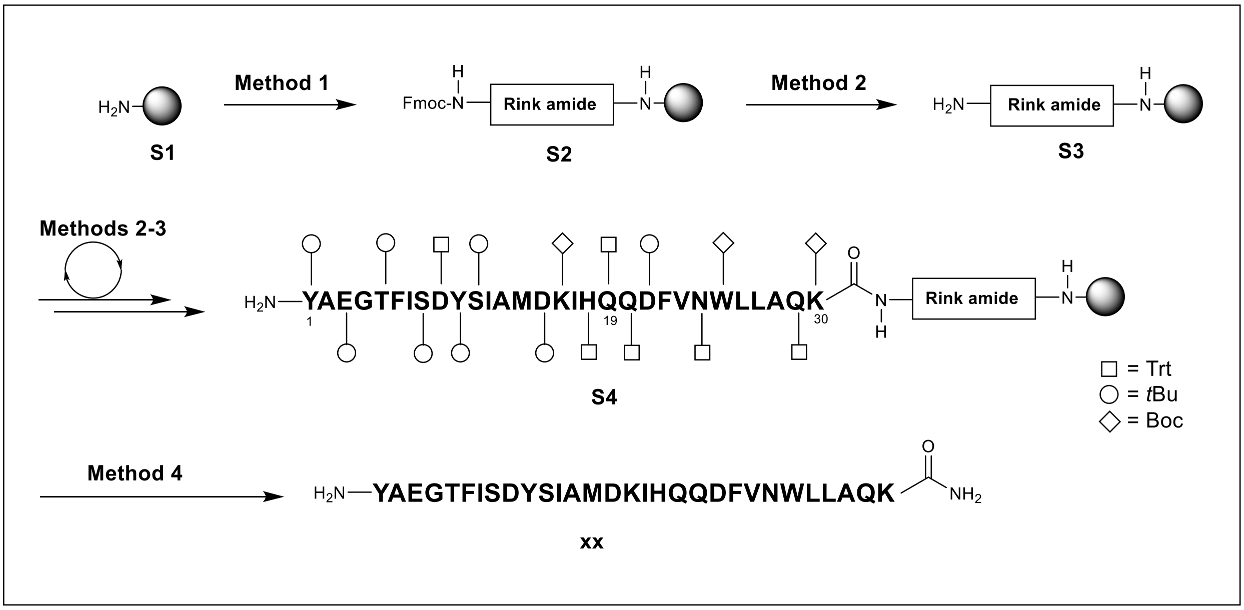

Scheme 1. Synthesis of GIP(1-30)

<u>Synthesis of GIP(1-30) peptide</u>

Fmoc-Rink amide was attached to aminomethyl TentaGel® S resin **S1** using **Method 1** followed by Fmoc-removal using **Method 2** to afford resin bound amine **S3**. Direct attachment of *C*-terminal amino acid, Fmoc-Lys(Boc)-OH to resin-bound Rink amide **S3** was achieved using **Method 3**. **Method 2** was used for all subsequent N^{α}-Fmoc removals where appropriate. Linear elongation of the peptide chain was achieved by coupling appropriate Fmoc-amino acids indicated in **Scheme S1** using **Method 3**. ^{19}Gln was coupled twice using fresh solutions of amino acid, HATU and DIPEA in DMF. Peptidyl resin **S4** was subjected to simultaneous global protecting group removal and resin cleavage using **Method 4**, affording crude peptide (300 mg). Crude peptide was purified batch-wise (10 mg of crude peptide) by semi-preparative RP-HPLC on a Phenomenex Gemini® C18 (5 µm, 10 × 250 mm) using a linear gradient of 5% to 95% over 90 min (*ca.* 1% B/min) with a flow rate of 4 mL/min. Fractions were collected at 0.2 min intervals and analysed by ESI-MS and RP-HPLC. Fractions identified with correct *m/z* were combined and lyophilised to afford GIP (1-30) peptide as a white amorphous solid. (9.7 mg, 27% yield based on 0.01 mmol scale)

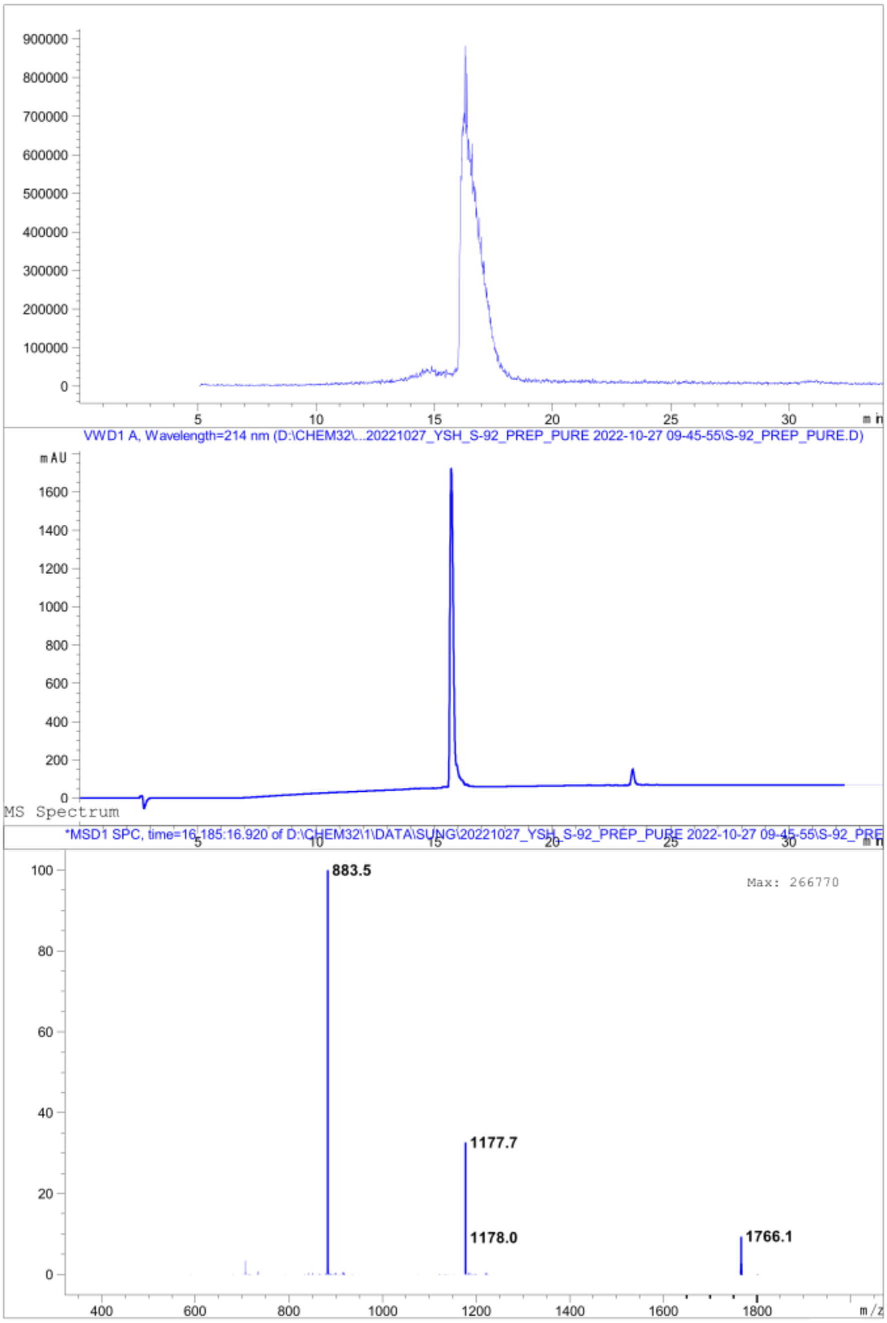

Figure SC1. LCMS profile of purified GIP (1-30) peptide, ion polarity positive operating at a nominal accelerating voltage of 70 eV. ESI-MS (*m/z* $[M+2H]^{2+}$ calcd: 1766.99; found 1766.1; $[M+3H]^{3+}$ calcd: 1178.33; found: 1177.7; $[M+4H]^{4+}$ calcd: 884.0; found: 883.5. Chromatographic separations were performed using an Agilent C3 analytical column (3.5 µm; 3.0 × 150 mm) and a linear gradient of 5-95% B over 30 min (*ca*. 3% B per min) at a flow rate of 0.3 mL/min. Buffer A: H_2O containing 0.1% formic acid (*v/v*); Buffer B: acetonitrile containing 0.1% formic acid (*v/v*).

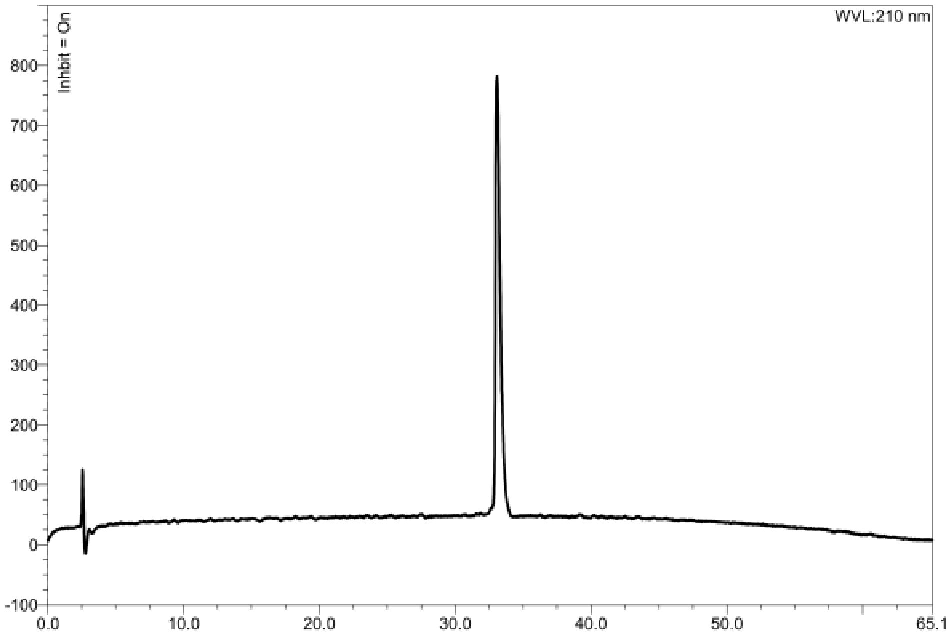

Figure SC2: HPLC profile of purified GIP (1-30) peptide. Chromatographic separations were performed using a Phenomenex Gemini® C18 (5 µm; 4.6 × 150 mm) and a linear gradient of 5-65% B over 60 min (*ca*. 1% B per min) at a flow rate of 1.0 mL/min. Buffer A: H_2O containing 0.1% TFA (*v/v*); Buffer B: acetonitrile containing 0.1% TFA (*v/v*).

Supplemental Results

```
                          10          20            30          40
GIP(1-42):    YAEGTFISDYSIAMDKIHQQDFVNWLLAQKGKKNDWKHNITQ
GIP(1-30):    YAEGTFISDYSIAMDKIHQQDFVNWLLAQK-NH2
Tirzepatide:  YXEGTFTSDYSIXLDKIAQKAFVQWLIAGGPSSGAPPPS
                                  └─C20 FATTY DIACID
```

Figure S1: Sequence of peptides used. X = Aib, 2-Aminoisobutyric acid.

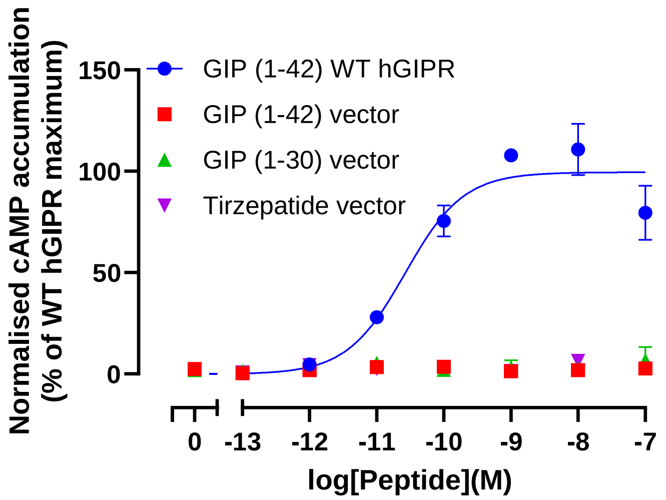

Figure S2: cAMP accumulation in vector (pcDNA3.1) transfected Cos7 cells. Data were normalized to the maximal cAMP produced by GIP(1-42) at the WT human GIP receptor and expressed as a percentage. Data points are the mean ± s.e.m of the combined data from three independent experiments.

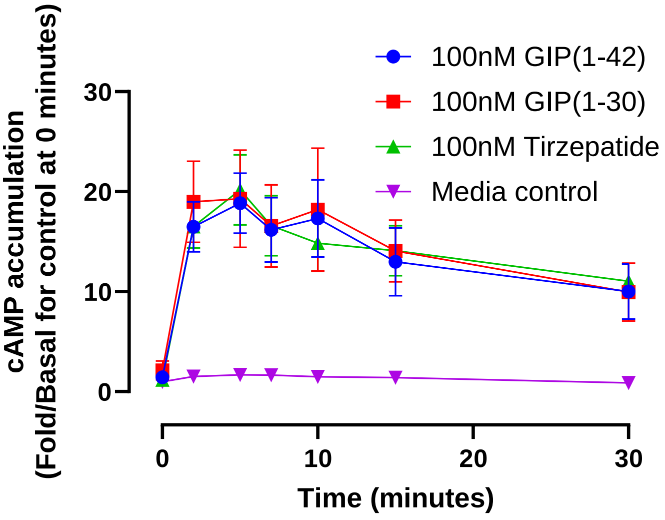

Figure S3: Human WT GIP receptor time course of cAMP accumulation by 100 nM GIP(1-42), GIP1-30), Tirzepatide, and media control in transfected Cos7 cells. Data are expressed as the fold change above the media-stimulated production of each signaling molecule. Data points are the mean ± s.e.m of the combined data from three independent experiments.

Supplemental Tables

Table S1: Summary of peptide E_{min} and E_{max} values at WT and E354Q GIP receptors in transfected Cos7 cells.

GIP Receptor	Peptide	cAMP (nM)		IP_1 (nM)		pAKT		pERK1/2		pCREB	
		E_{min}	E_{max}	E_{min}	E_{max}	E_{min}	E_{max}	E_{min}	E_{max}	E_{min}	E_{max}
**WT**	**GIP(1-42)**	4.62 ± 0.71	34.2 ± 7.76	124 ± 26.8	245 ± 31.5	2176 ± 571	9186 ± 3379	43070 ± 6118	167768 ± 5869	25342 ± 8746	41709 ± 17541
	**GIP(1-30)**	4.52 ± 0.62	32.2 ± 8.97	139 ± 25.0	263 ± 50.0	2211 ± 443	8828 ± 3104	42093 ± 4410	177161 ± 8832	26907 ± 9741	45007 ± 20307
	**Tirzepatide**	4.52 ± 0.54	33.4 ± 8.46	118 ± 22.3	191 ± 18.1	2003 ± 528	8518 ± 3287	38531 ± 6580	148142 ± 2760	24314 ± 9065	41831 ± 19059
**E354Q**	**GIP(1-42)**	5.15 ± 0.79	35.5 ± 8.91	111 ± 29.5	178 ± 26.4	2181 ± 723	6372 ± 2555	44214 ± 12888	138058 ± 15843	25769 ± 12478	40843 ± 20525
	**GIP(1-30)**	5.00 ± 0.97	32.8 ± 7.78	115 ± 29.5	186 ± 24.3	2156 ± 483	5634 ±1817	43582 ± 12773	133276 ± 17603	27863 ± 13062	40158 ± 19643
	**Tirzepatide**	5.25 ± 0.74	32.5 ± 7.88	118 ± 27.8	167 ± 21.4	2100 ± 738	6250 ± 2787	40156 ± 13007	134832 ± 27518	27451 ± 13895	40839 ± 22194

Data are the mean ± s.e.m of the combined data from 3 (pAKT, pERK1/2, pCREB) or 5 (cAMP, IP_1) independent experiments. E_{min} and E_{max} values are derived from the bottom and top of three or four-parameter curve fit. Values for cAMP and IP1 are expressed in nM, and values for pAKT, pERK1/2 and pCREB are arbitrary units.

Table S2: Summary of peptide relative efficacy (Δlog(τ/KA)) and biased agonism (ΔΔlog(τ/KA)) values at WT and E354Q GIP receptors in transfected Cos7 cells.

GIP Receptor	Peptide	cAMP		IP_1		pAKT		pERK1/2		pCREB	
		$\Delta\log(\tau/K_A)$	$\Delta\Delta\log(\tau/K_A)$	$\Delta\log(\tau/K_A)$	$\Delta\Delta\log(\tau/K_A)$	$\Delta\log(\tau/K_A)$	$\Delta\Delta\log(\tau/K_A)$	$\Delta\log(\tau/K_A)$	$\Delta\Delta\log(\tau/K_A)$	$\Delta\log(\tau/K_A)$	$\Delta\Delta\log(\tau/K_A)$
**WT**	**GIP(1-42)**	0.00 ± 0.12	0.00 ± 0.17	0.00 ± 0.10	0.00 ± 0.15	0.00 ± 0.16	0.00 ± 0.20	0.00 ± 0.25	0.00 ± 0.28	0.00 ± 0.32	0.00 ± 0.34
	**GIP(1-30)**	0.10 ± 0.12	0.00 ± 0.17	0.18 ± 0.11	0.08 ± 0.16	0.09 ± 0.12	-0.01 ± 0.17	-0.05 ± 0.22	-0.15 ± 0.25	0.23 ± 0.30	0.13 ± 0.33
	**Tirzepatide**	**-0.75 ± 0.32***	0.00 ± 0.45	**-0.87 ± 0.29***	-0.12 ± 0.43	**-1.16 ± 0.34***	-0.41 ± 0.46	-1.13 ± 0.43	-0.38 ± 0.54	-0.81 ± 0.48	-0.06 ± 0.58
**E354Q**	**GIP(1-42)**	0.00 ± 0.25	0.00 ± 0.36	0.00 ± 0.14	0.00 ± 0.29	0.00 ± 0.02	0.00 ± 0.25	0.00 ± 0.19	0.00 ± 0.32	0.00 ± 0.34	0.00 ± 0.42
	**GIP(1-30)**	0.19 ± 0.18	0.00 ± 0.26	-0.14 ± 0.10	-0.33 ± 0.21	0.01 ± 0.10	-0.18 ± 0.21	0.23 ± 0.30	0.04 ± 0.35	1.04 ± 0.48	0.85 ± 0.51
	**Tirzepatide**	**-0.90 ± 0.43***	0.00 ± 0.60	**-1.18 ± 0.34***	-0.28 ± 0.55	**-1.32 ± 0.19***	-0.42 ± 0.47	**-1.30 ± 0.38***	-0.40 ± 0.57	-1.27 ± 0.51	-0.37 ± 0.66

Data were analyzed using the Operational model of allosterism and are mean ± s.e.m of the combined data from 3 (pAKT, pERK1/2, pCREB) or 5 (cAMP, IP_1) independent experiments. Statistical differences in relative efficacy between different agonists at the same receptor compared to GIP(1-42) or biased differences for the same agonist between different pathways compared to cAMP accumulation (biased agonism) were determined using a one-way ANOVA with post-hoc Dunnett's test on the $\Delta\Delta\log(\tau/K_A)$ values. *$p < 0.05$ compared to GIP(1-42) for relative efficacy ($\Delta\log(\tau/K_A)$) of each agonist at each pathway at each receptor. No significant differences in biased agonism ($\Delta\Delta\log(\tau/K_A)$) observed for agonists across pathways.
